# Raman Spectroscopic Study of TiO_2_ Nanoparticles’ Effects on the Hemoglobin State in Individual Red Blood Cells

**DOI:** 10.3390/ma14205920

**Published:** 2021-10-09

**Authors:** Elena Perevedentseva, Yu-Chung Lin, Artashes Karmenyan, Kuan-Ting Wu, Andrei Lugovtsov, Evgeny Shirshin, Alexander Priezzhev, Chia-Liang Cheng

**Affiliations:** 1 Department of Physics, National Dong Hwa University, Hualien 974301, Taiwan; 810014103@gms.ndhu.edu.tw (Y.-C.L.); artashes@gms.ndhu.edu.tw (A.K.); kkfire1234567@gmail.com (K.-T.W.); 2 P.N. Lebedev Physical Institute of Russian Academy of Sciences, 119991 Moscow, Russia; 3 Department of Physics, Moscow State University, 119991 Moscow, Russia; anlug1@gmail.com (A.L.); eshirshin@gmail.com (E.S.); avp2@mail.ru (A.P.)

**Keywords:** red blood cell, hemoglobin, titanium dioxide nanoparticles, Raman spectroscopy, Raman mapping

## Abstract

Titanium dioxide (TiO_2_) is considered to be a nontoxic material and is widely used in a number of everyday products, such as sunscreen. TiO_2_ nanoparticles (NP) are also considered as prospective agents for photodynamic therapy and drug delivery. These applications require an understanding of the potential effects of TiO_2_ on the blood system and its components upon administration. In the presented work, we analyze the interaction of TiO_2_ nanoparticles of different crystal phases (anatase and rutile) with individual rat Red Blood Cells (RBC) and the TiO_2_ influence on the oxygenation state and functionality of RBC, estimated via analysis of Raman spectra of Hemoglobin (Hb) and their distribution along individual RBC. Raman spectral signals also allow localization of the TiO_2_ NP on the RBC. No penetration of the NP inside RBC was observed; however, both kinds of TiO_2_ NP adsorbed on the RBC membrane can affect the Hb state. Mechanisms involving the NP–membrane–Hb interaction, resulting in partial deoxygenation of Hb and TiO_2_ photothermal effect on Hb under Raman laser excitation, are suggested. The possible influence on the safety of TiO_2_ use in advanced medical application, especially on the safety and efficiency of photothermal therapy, is discussed.

## 1. Introduction

Current perspectives on developing nanoparticles’ (NP) applications for theranostics [1,2] require an understanding of NP interaction with, and potential effects on, the blood system and its components upon administration via injection. NP can also enter the blood stream after administration through breath, dermal deposition, and absorption in the gastrointestinal system. The interaction of various NP with blood and blood components has been studied. The state and functionality of the blood is determined in a significant degree by the state and functionality of red blood cells (RBC). Thus, one of the important criteria for NP use is how the NP can interact with RBC and affect the RBC functionalities. The influence of different kinds of NP on RBC under various conditions was studied, including carbon [3,4,5], silica [6,7], gold and silver [8,9,10,11], Fe_3_O_4_ [12] NP, and others [8]; mechanisms of interaction and the conditions of safe application were discussed. Although among other NP, titanium dioxide (TiO_2_) is often considered as a nontoxic material and is widely used in everyday products of pharmaceutics, cosmetics, and food industries, the problem of its safety when interacting with blood becomes relevant due to current research concerning applications of TiO_2_ NP for photodynamic therapy and drug delivery [13].

The interaction of TiO_2_ with RBC has been studied in a number of works [5,8,14,15,16,17,18], and in most of them different kinds of damaging effects were observed, from morphology abnormalities [17], hemolysis [14], membrane properties modification [18], and increasing procoagulant activity of RBC [15], up to induced cell death, genotoxicity, and inflammation [5,19], which depended on internalization pathways, ROS production, and the abnormal interaction of proteins [5]. The effect of TiO_2_ NP on blood was also observed in in vivo studies [20]. Therefore, TiO_2_ NP exhibit potential toxicity, and an understanding is needed of how and under which conditions TiO_2_ can be used to avoid fatal effects or, on the other hand, to induce toxicity for nanomedical purposes. 

The protein hemoglobin (Hb) contained in RBC is responsible for the function of blood gas transportation. Violations or modifications of this function by NP can be observed [3,4,10] and need to be controlled. To investigate these effects, noncontact and noninvasive means are essential. For this purpose, Raman spectroscopy is a suitable tool for analysis of the Hb function and its dynamics due to a selective enhancement of the Raman active vibrations in different forms of Hb [3,4,10,21,22,23]. Additionally, Raman mapping allows for a direct observation of the distribution of Hb in different states. It has been applied for analyzing the formation of Hb derivatives in RBC under varying conditions [21,24], studying the process of erythrophagocytosis of senescent RBC [23], and for diagnosing malaria [24].

Raman spectroscopy and Raman mapping were shown to be effective tools for analysis of NP–RBC interaction. Previously, the effects of NP on the oxygenation state of individual RBC were studied for nanodiamond (ND), and it was shown that the adhesion of ND on the RBC membrane affects the cell functioning [3,4,18], but safe conditions for applications can be selected. NDs of different sizes stick to the RBC membrane, slightly affect the deoxygenation degree of RBC, and influence the oxygenation–deoxygenation dynamics of RBC in size-, concentration-, and surface-chemistry-dependent ways [3,4]. More serious disturbance effects were observed in the study of Au and Ag NP interaction with RBC [10]. Increasing hemoglobin deoxygenation was observed in both Au and Ag NP-treated RBC. These studies suggest that the adhesion of NP on the cell membrane causes an imbalance in RBC functioning and cell damage [10,11]. Independent of size and chemical composition, NP penetration into RBC was not observed in these works. In contrast to this, the penetration through the RBC membrane of NP and their aggregates with sizes less than 200 nm was observed using fluorescence microscopy for polymer (with fluorescent dye) NP and via electron microscopy for gold and TiO_2_ anatase NP after incubation for 4–48 h [8]. Larger particles or aggregates were found stuck to the RBC surface. 

In the present work, we analyze the interaction of TiO_2_ NP of different crystal phases (anatase and rutile) with individual rat RBC. The oxygenation state and functionality of RBC is estimated via analysis of Raman spectra from the Hb Raman mapping of an individual RBC. Raman mapping allows observation of the distribution of Hb in different oxygenation states in the studied RBC. Simultaneously, Raman spectra allow localization of the TiO_2_ NP on the RBC. In this work, no penetration of the NP inside the RBC was observed; however, both kinds of TiO_2_ NP were adsorbed on the RBC membrane, which may alter the oxygenation degree in the conditions when RBC should be oxygenated or, in general, could affect the Hb form. The effects of rutile and anatase TiO_2_ NP are compared. Possible mechanisms of influence of the membrane-localized TiO_2_ NP on Hb are discussed.

## 2. Materials and Methods

To estimate the effect of TiO_2_ nanoparticles on the RBC oxy/deoxy state, two kinds of TiO_2_ NP with predominant rutile, TiO_2_(r) (Sigma, Saint Louis, MO, USA), and anatase, TiO_2_(a), (UV100 Hombikat, Sachtleben Chemie GmbH, Duisburg, Germany) structures were used. The nominal size of TiO_2_(r) particles was ≤100 nm, and that of TiO_2_(a) was about 10 nm. In the experiments, the particles’ size and ξ-potential were estimated using the dynamic light scattering method (DLS) with a Zetasizer Nano ZS (Malvern Instruments, Malvern, UK). NP were suspended in ultrapure MilliQ water (Type 1, Direct-Q 3UV, Merck-Millipore, Burlington, MA, USA) at pH 6 and in PBS (pH 7.3) in concentration 0.1 mg/mL. Absorption spectra were measured with a UV–visible spectrometer to confirm high absorption from UV up to 450 nm range. Structural phases of TiO_2_ NP were confirmed by Raman spectra measurements with an α-SNOM Raman microspectrometer (Witec, Ulm, Germany), with excitation by an Ar ion laser with 488 nm wavelength.

For the rat red blood cell (RBC) samples, 3 mL of whole blood was withdrawn from the tails of Wistar rats and transferred into EDTA-covered tubes. The research methods were approved by the Animal Care and Use Committee of National Dong Hwa University (Approval ID100004). The rats were narcotized for the experiments. RBC samples were prepared and treated with NP as described previously [3,4]. In short, the RBC were separated from the fresh whole blood using centrifugation, repeatedly washed with standard phosphate buffer saline (pH 7.4), then RBC mass was diluted with PBS at the ratio of 5:1000 µL (RBC:PBS). The spectra of RBC in an oxygenated state were measured in ambient conditions and after nitrogen gas purging for deoxygenated RBC.

The TiO_2_ powders were diluted in PBS at 33 mg/mL concentration. The obtained suspension was added to the RBC:PBS sample and coincubated for 1 h with the final concentration of TiO_2_ ~20 µg/mL in the prepared sample. The prepared sample suspensions of RBC with TiO_2_ powders and control RBC without TiO_2_ were placed on a Si substrate for Raman measurements. The samples were at ambient atmospheric pressure, so RBC were oxygenated [3,4]. Raman spectra were measured and the selected Raman signal intensity mapping was performed with α-SNOM Raman microspectrometer (Witec, Ulm, Germany), with excitation by an Ar ion laser of 488 nm wavelength, objective ×50, power ~0.3 mW at the output, scanning time 150–220 s in ambient atmosphere conditions. Raman mapping of 15–20 RBCs from each sample were performed. Every mapping contained 24 × 24 spectra which were analyzed using software of Witec α-SNOM ScanCntr Spectroscopy Plus 1.34 (Witec, Ulm, Germany) and then with Origin 9.1 (OriginLab, Northampton, MA, USA).

## 3. Results

### 3.1. TiO_2_ Particles Characterization

The size and ζ-potential of rutile TiO_2_(r) and anatase TiO_2_(a) phases NP suspended in ultrapure MilliQ water were measured. The ζ-potentials were found to be 15.5 ± 0.37 mV for anatase NP and 11.1 ± 0.36 mV for rutile. The size of TiO_2_(r) at that condition showed a narrow distribution with a maximum near 154.4 ± 16.1 nm; TiO_2_(a) revealed two fractions, one centered near size 136 nm and the other, characterizing the aggregates, with an average size of 448.1 ± 27.3 nm. When dissolved in PBS (pH 7.3), the measured values of ζ-potentials became negative and equal to −23.4 ± 2.0 and −25.2 ± 1.43 mV, respectively for the TiO_2_(r) and anatase TiO_2_(a). From the particle size distribution, the TiO_2_ in PBS aggregated or agglomerated and formed a wide distribution with the hydrodynamic size up to one micron (data not shown); however, some crucially decreased fraction of small particles or small aggregates still exists. We deliberately did not remove the larger aggregates, as we wanted to examine the more practical application when RBC encounter all kinds of TiO_2_ aggregates, especially in PBS. The particles’ agglomeration significantly increased in PBS in comparison with water, as was shown for TiO_2_ NP in solutions with high ionic strength at pH 7.3 [25]. In such studies, the primary size of the TiO_2_ NP, usually tens of nm, together with the crystallinity, played a role in the properties of agglomerates, and the properties inherent in nanoscale particles could also manifest at TiO_2_ interaction with cells. The observed average hydrodynamic size of agglomerates reached 751.2 ± 48.8 nm (rutile) and 673 ± 29.0 nm (anatase). The agglomeration and changes in ζ-potential may have happened due to the influence of PBS components, as has been shown previously [26], where ions such as Cl^−^, Na^+^, and PO_4_^−3^ from PBS can affect the particle surface properties and stabilization of TiO_2_ NP suspension. 

**Figure 1:**
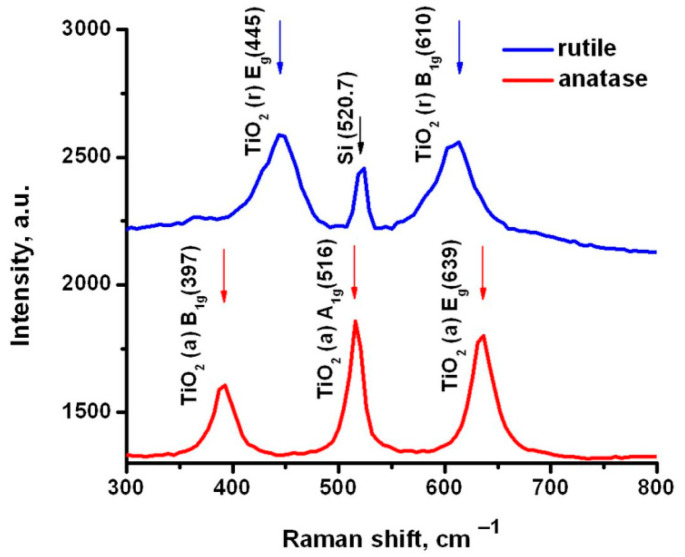
Raman spectra of Rutile TiO_2_(r) and Anatase TiO_2_(a) NP with 488 nm wavelength laser excitation. The peaks, positions, and modes are marked according to literature values.

Typical Raman spectra of the studied TiO_2_ powders are shown in Figure 1. Characteristic peaks were observed and agreed with literature values [27]. The spectra confirm the structure of two kinds of TiO_2_ NP used in this work. These observed characteristic peaks were used as markers to localize TiO_2_ in the Raman mappings of RBC at their interaction with TiO_2_.

### 3.2. Raman Analysis of the RBC Oxygenation State

**Figure 2:**
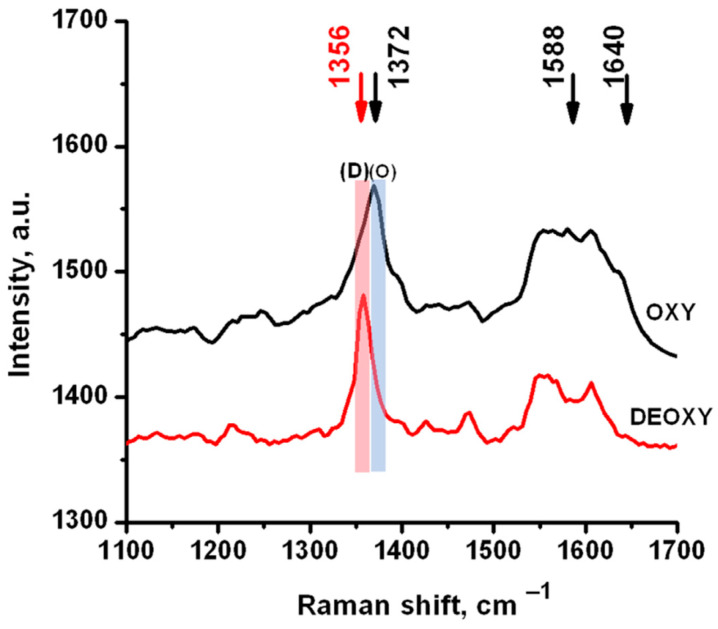
Raman spectra of oxyHb and deoxyHb, measured in oxygenated and deoxygenated RBC with 488 nm wavelength laser excitation. (O) and (D) mark the wavenumber ranges for mapping of **ν**_4_ band: oxyHb (1365–1380 cm^−1^) and deoxyHb (1350–1360 cm^−1^), respectively. Peaks for oxyHb 1588 cm^−1^ (**ν**_37_) and 1640 cm^−1^ (**ν**_10_) are also shown. Peak assignment according to [21].

The red blood cells’ main component is Hb, the iron-containing oxygen transporting protein. Raman spectroscopy is used for studies of Hb and RBC; aside from its low-invasive nature, it allows for the simultaneous identification of oxygenated and deoxygenated states of Hb. Hemoglobin’s Raman spectrum is strong and complex but with fingerprints from heme (the active center of Hb) and protein units [21,22,24]. The spectra of oxygenated and deoxygenated RBC are shown in Figure 2. These spectra were measured from individual RBC with 488 nm wavelength laser excitation. Raman vibrations in different oxygenation states exhibited obvious changes in the oxidation state sensitive region (1350 to 1380 cm^−1^, **ν**_4_ band), spin state sensitive region (1500 to 1650 cm^−1^, **ν**_19_, **ν**_37_, and **ν**_10_ bands), and the C-H deformation region (1200 to 1250 cm^−1^), followed by T ↔ R (tense–relaxed) transition in the Hb molecule in the oxygenation–deoxygenation process [22,28,29]. Based on the light absorption spectrum of oxyHb and deoxyHb, excitation can be selected close to the absorption bands to result in selective enhancement of resonance Raman peaks [30]. The Soret band maxima of oxy- and deoxy-Hb were 415 and 430 nm, respectively, and it has been shown that at 488 nm wavelength laser excitation, clear spectral changes in the 1350–1370 cm^−1^ (**ν**_4_ band) can be observed [28]. This band was assigned to the symmetrical pyrrole half-ring stretching vibration and the shift from 1376 cm^−1^ to 1358 cm^−1^ was demonstrated as a marker of deoxygenation at resonance Raman study of the oxygenation–deoxygenation process [30]; the peak’s intensity ratio was proportional to the portion of oxygenated Hb [4,30].

**Figure 3:**
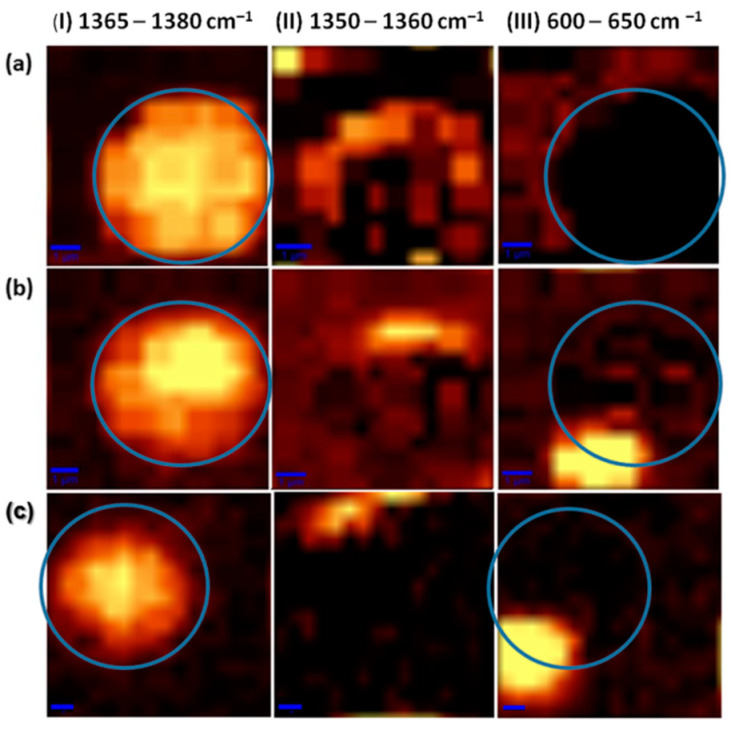
Raman mapping of individual RBC: (**a**) control RBC; (**b**) RBC with TiO_2_ (Rutile); and (**c**) RBC with TiO_2_ (Anatase). (I) mapping in the range of 1365–1380 cm^−1^ shows distribution of oxyHb; (II) mapping in the range 1350–1360 cm^−1^ shows the distribution of deoxyHb; and (III) mapping in the range 600–650 cm^−1^ shows the distribution of the TiO_2_ peaks only in the TiO_2_-treated RBC. The spectra were collected at ambient conditions, 488 nm wavelength laser excitation; 24 × 24 pixels. Scale bar is 1 μm. The RBC contour in mapping of TiO_2_ in (III) is marked according to the cell mapping in (I).

Typical spectra for oxyHb and deoxyHb are displayed in Figure 2, with the markers for the Raman mapping. The Raman mapping of RBC treated with TiO_2_ was performed and compared with the control of the untreated RBC. Figure 3 shows the typical mapping of control RBC (Figure 3a) and RBC treated with TiO_2_(r) (Figure 3b,c). Raman mapping illustrated the spatial distribution of Raman signal intensity along the sample; the distribution of oxyHb was mapped via Raman peak with a maximum in the 1365–1380 cm^−1^ range (O in Figure 2) and distribution of deoxyHb via a peak in the 1350–1360 cm^−1^ range (D in Figure 2). TiO_2_ was visualized via mapping of Raman signal in the 600–650 cm^-1^ range where A_1g_ band of Rutile (610 cm^−1^) and E_g_ band of Anatase (639 cm^-1^) were revealing, peaks shown in Figure 1. Note that the measurements were performed in ambient conditions; thus, the RBC should be mostly oxygenated. The spectra measured from the TiO_2_-treated and untreated RBC allowed us to observe and discuss the effect of TiO_2_ NP on oxygenated RBC, while mapping of the intensity of Raman signal in characteristic RBC (Hb) and TiO_2_ ranges allowed, correspondingly, visualizing the relative distributions of oxygenated oxyHb (Figure 3-I), deoxygenated deoxyHb (Figure 3-II), and TiO_2_ localization at interaction with the RBC (Figure 3b,c-III) in comparison with the control (Figure 3a-III). Note that TiO_2_ is not fluorescent and cannot be easily observed with the methods of laser fluorescence scanning microscopy, while Raman mapping serves as an alternative means of TiO_2_ visualization.

In the images of a control cell (Figure 3a) the part of oxygenated Hb was localized in the center of the RBC (Figure 3a-I), the relatively deoxygenated Hb was distributed on the peripheral part of RBC (Figure 3a-II). In general, a similar distribution was observed for mapping of RBCs treated with TiO_2_. In Figure 3b,c, Raman mappings of an RBC treated with TiO_2_(r) and TiO_2_(a) are presented, and the maps of relatively oxygenated Hb (Figure 3b,c-I) and relatively deoxygenated Hb (Figure 3b,c-II) were added with a distribution of TiO_2_ (Figure 3b,c-III). Well-detectable aggregates of TiO_2_ localized on the outer surface of the RBC membrane were observed in both cases of using Anatase and Rutile particles. In spite of a strong Raman signal of TiO_2_, we did not observe any NP which could be considered localized inside the cell.

**Figure 4:**
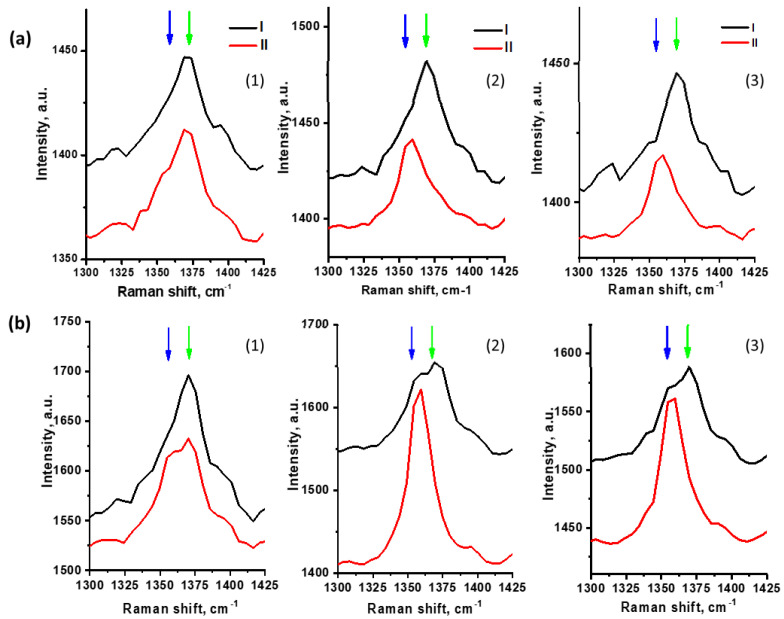
Characteristic Raman spectra of individual RBCs extended in the 1300–1425 cm^−1^ range. Examples for (**a**) the most oxygenated RBCs; and (**b**) the most deoxygenated RBCs. For every RBC, the spectra demonstrate the higher (I) and lower (II) oxygenation states. (1) Control RBC; (2) RBC treated with TiO_2_(r); and (3) RBC treated with TiO_2_(a). Blue and green arrows mark the **ν_4_** band at 1356 cm^−1^ and 1372 cm^−1^, respectively.

Both Rutile and Anatase NP were tested before, and the toxicity of Rutile and Anatase could differ. In different studies, the results were controversial and depended significantly on the experimental conditions [31,32,33,34]. In order to discuss the possible effect of both kinds of TiO_2_ NP on RBC, the spectra were analyzed focusing on the areas of maximum oxygenation (as shown in Figure 3-I) and maximum deoxygenation (as in Figure 3-II) in the Raman maps of each studied individual RBC. In Figure 4, the spectra of individual RBCs in a narrow range containing the band **ν**_4_ are shown. A large number of individual cells was analyzed, the variability in Hb oxygenation state distribution along one cell and an average oxygenation degree for the different cells was observed. The spectra for each analyzed RBC were selected to demonstrate the highest (I) and lowest (II) oxygenation degree, which could be observed in the same individual cell (Figure 4a). The spectra in Figure 4a,b were measured from similar areas of two different cells and show the oxygenation degree of variability between cells. Figure 4(1) shows spectra for control untreated RBCs, while Figure 4(2,3) show spectra for RBCs treated with Rutile TiO_2_(r) and TiO_2_(a), respectively. 

Figure 4 shows the data for cells indicating the largest difference found in the oxygenation degree inside one cell differs for untreated and TiO_2_-treated RBC. The spectra shown for higher (I) and lower (II) oxygenation states of control RBC (Figure 4(1)) with the maximum at 1372 cm^−1^ indicated mostly oxygenated Hb. Only a weakly expressed shoulder (arising from the peak at 1356 cm^−1^) was observed in the spectra (II) both in Figure 4a(1),b(1), so the content of deoxygenated Hb can be considered very low. However, this low content of particularly deoxygenated Hb was still observable with Raman mapping in Figure 3a-II.

As the Raman peak intensity directly depends on the number of oscillators, the oxygenation degree SO_2_ can be considered proportional to the ratio of intensities **I_1372_** and **I_1356_** [4,30], and can be expressed as SO_2_ = A × **I_1372_**/(**I_1372_** + **I_1356_**) + B, where **I** is the peak intensity at the corresponding wavenumber, and A and B are coefficients, which have to be determined via a calibration with independent oximetry measurements [28].

**Figure 5:**
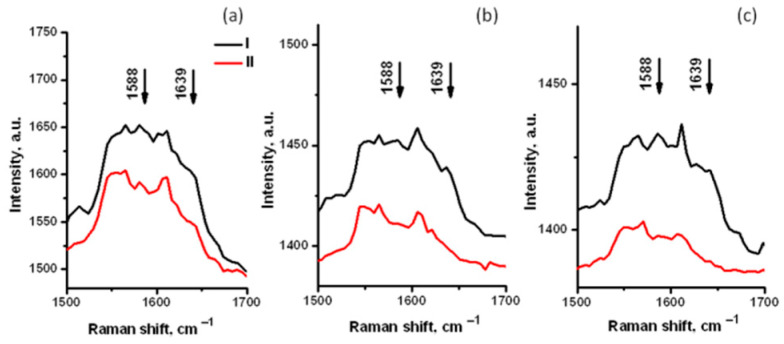
Characteristic Raman spectra measured in the most oxygenated and in the most deoxygenated areas of an individual RBC: (**a**) control; (**b**) RBC treated with TiO_2_(r); and (**c**) RBC treated with TiO_2_(a). Spectra are extended in the 1500–1700 cm^−1^ range; oxygenation state marker bands are shown. For every RBC, the spectra demonstrate higher (I) and lower (II) oxygenation states.

**Figure 6:**
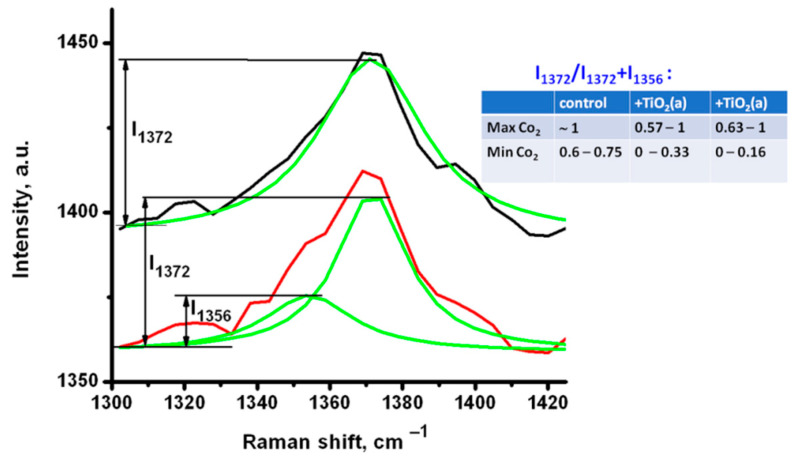
Deconvolution of characteristic Raman spectra of the most oxygenated Hb (I) and the most deoxygenated Hb (II) in an individual RBC; spectra deconvolution using Lorentzian fitting is shown in green. Inset: the variations of intensities ratio for control RBC and RBC treated with TiO_2_ are estimated numerically.

Figure 4a(1),b(1) show that the control RBC were predominantly oxygenated, as expected in ambient conditions. **I_1372_**/(**I_1372_** + **I_1356_**) can be considered close to 1 in the RBC center (as in Figure 3a), and it decreased in the thin layer on the periphery in the cell (Figure 3b). Figure 4(2,3) show the spectra for RBC treated with different kinds of TiO_2_. In contrast to Figure 4(1), in Figure 4a(2),a(3), oxyHb in the center of the cell, with a ratio **I_1372_**/(**I_1372_**+ **I_1356_**) close to 1, coexisted with significantly deoxygenated Hb on the cell periphery. An even lower oxygenation degree was estimated along the whole cells, presented by spectra in Figure 4b(2),b(3), where the central part of RBC contained only particularly oxygenated Hb, with both well-observable peaks at 1372 and 1356 cm^−1^, while the periphery revealed mostly deoxyHb. Thus, the content of deoxyHb was observed to be higher in both TiO_2_-treated cells than in the control. Spectra extended in the spin-sensitive range (Figure 5) also confirmed a decrease in oxyHb markers (**ν_37_** at 1588 cm^−1^ and **ν_10_** at 1640 cm^−^^1^) for TiO_2_-treated RBC (Figure 5b,c) in comparison with untreated cells (Figure 5a). This means that TiO_2_ adsorbed on the RBC membrane may affect Hb oxygenation degree in the air environment when RBC should be oxygenated. Analyzing the variability of TiO_2_ effect on oxygenated RBC, no difference between the Rutile and Anatase particles was observed. Maxima for **ν**_4_ in oxygenated and deoxygenated states were determined via the spectra deconvolution (Figure 6), and the value **I_1372_**/(**I_1372_** + **I_1356_**) characterizing the variability of RBC oxygenation for untreated and treated RBC is presented in the inset of Figure 6.

## 4. Discussion

In previous studies, a shift of the **ν**_4_ band in the process of Hb oxygenation was observed [30], and it was demonstrated that the ratio of intensities of the peaks at 1372 cm^−1^ and 1356 cm^−1^ could be used as a marker of the oxygenation state of Hb [10,21,30]. Additionally, in Raman mapping, we observed the inhomogeneous distribution of Hb with various oxygenation degree values along an individual RBC, and analyzed how the adsorption of TiO_2_ on the RBC membrane affected this distribution. The predominant localization of oxygenated Hb is in the RBC center (Figure 3a-I), while a small fraction of partially deoxygenated Hb is distributed on the peripheral part of the control RBC (Figure 3a-II). It is consistent with the studies of deoxyHb binding on RBC membrane as a result of electrostatic interactions, covalent association with the membrane components by disulfide bonds, and adsorption to membrane lipids via hydrophobic interactions [35]. In particular, the reversible association of deoxyHb with the RBC membrane via interaction with band 3 protein provides an active role of the membrane in RBC gases’ transport function [35]. It is considered a “molecular switch” mechanism for regulating RBC biology (metabolic and transport processes, signal transduction, etc.) [36]. Similarly, RBCs treated with both kinds of TiO_2_ demonstrated the peripheral localization of some portion of deoxyHb and the central localization of Hb with a higher oxygenation degree (Figure 3b, for TiO_2_(r) and Figure 3c for TiO_2_(a)). However, the oxygenation degree of the whole TiO_2_-treated RBC was decreased as is shown in the inset of Figure 6. 

As the plasma membrane of RBC plays a fundamental role in the oxygen (and other gases) transport by RBC and in maintaining RBC metabolism [37], the membrane structural changes, which can be created by adsorbed NP aggregates, can affect the RBC state [3,4,10,11,18]. Observed TiO_2_ aggregates or agglomerates on the RBC membrane agreed, e.g., a previous study showed TiO_2_ particles sticking to RBC membrane regardless of their surface charge [18]. It suggests the mostly nonelecrostatic nature of the NP–membrane adhesion interaction; however, it is not clear, and more detailed studies are needed to confirm. The membrane permeability for oxygen is determined by the membrane molecules’ mobility; thus, the interaction between the membrane and adhered NP aggregates or agglomerates can affect the conditions for the gas molecules’ transmembrane diffusion and for Hb adsorption on the membrane. Additionally, as was shown [10], the interaction with adsorbed NP may create an obstacle for the exchange of ions and polar molecules via protein channels and also due to production of reactive oxygen species [14] and lipid peroxidation. These reasons can lead to altering cell membrane properties, pH imbalances inside the cells, and finally can affect the oxygen binding affinity of Hb. As a result, similar deoxygenation under NP treatment has been observed [10] for RBC trapped with optical tweezers and treated with Ag or Au NP. 

Additionally, using an excitation laser with 488 nm wavelength, we needed to consider the probability of laser effects directly on Hb [38], such as photodegradation of Hb (denaturation, hemichrome formation, and potential aggregation) in the process of Raman measurements. Some researchers suggest that the position of the **ν_4_** peak can indicate the iron ion transition to the ferric form [39], and this is considered as one of the markers of the Hb photodamage [40]. This effect is pronounced in the presence of both kinds of TiO_2_ in comparison with control. Thus, this combined effect is facilitated by the oxidative stress due to photocatalytic and phototoxic properties of TiO_2_ at the excitation close to the TiO_2_ absorption range [41]. Most of the resulting Hb forms contained the ferric heme iron and could hardly be distinguished from oxyHb. However, comparison of the spectra in Figure 4 and Figure 5 shows that the position of the **ν_4_** peak at 1372 cm^−1^ correlated with the presence of other peaks characterizing oxyHb (**ν_37_** 1588 cm^−1^ and **ν_10_** 1640 cm^−1^), while **ν_4_** observed at 1356 cm^−1^ correlated with their decreasing. This decrease in intensity of **ν_37_** and **ν_10_** peaks allows us to conclude that particular deoxygenation of Hb takes place, rather than Hb photodegradation. We suggest TiO_2_ affects Hb state through affecting the membrane properties and stimulats Hb deoxygenation.. We cannot exclude also the input of photodissociation of Hb, also resulting in the observation of higher content of deoxyHb. We suggest also that, together with influence on Hb through affecting the membrane properties and stimulating Hb deoxygenation membrane-associated TiO_2_, aggregates/agglomerates can promote photo or photothermal dissociation of the oxyHb. 

Use of TiO_2_ NP for nanotherapy is currently widely discussed [13]. The limitations of their application as photosensitizers for photodynamic therapy are discussed in terms of possible side-effects, but they are considered nontoxic for their use in drug delivery or for ultrasound activation of ROS formation for anticancer treatment [42,43]. However, significant and various detrimental influences of TiO_2_ NP on blood and blood components are demonstrated, and understanding these effects and studying these mechanisms, including the nonphotoactivated ones, is necessary. 

In conclusion, the TiO_2_ effect on RBC was observed, resulting in a decrease in the oxygenation degree of RBC. We suggest that TiO_2_ affects Hb oxygenation state via altering RBC membrane properties. The disturbing treatment of the laser in the presence of TiO_2_ is also probable, resulting in oxygen photodissociation of Hb. These effects should be considered in the development of the method of TiO_2_ NP theranostic applications which imply NP interaction with blood.

## Data Availability

The data presented in this study are available within the article.

## References

[1] Urbán P., Liptrott N.J., Bremer S. (2019). Overview of the blood compatibility of nanomedicines: A trend analysis of in vitro and in vivo studies. WIREs Nanomed. Nanobiotechnol..

[2] Zhang N., Wei M.Y., Ma Q. (2019). Nanomedicines: A Potential Treatment for Blood Disorder Diseases. Front. Bioeng. Biotechnol..

[3] Lin Y.C., Tsai L.W., Perevedentseva E., Chang H.H., Lin C.H., Sun D.S., Lugovtsov A., Priezzhev A., Jani M., Cheng C.L. (2012). The influence of nanodiamond on the oxygenation states and micro rheological properties of human red blood cells in vitro. J. Biomed. Opt..

[4] Tsai L.W., Lin Y.C., Perevedentseva E., Lugovtsov A., Priezzhev A., Cheng C.L. (2016). Nanodiamonds for Medical Applications: Interaction with Blood in Vitro and in Vivo. Int. J. Mol. Sci..

[5] Boland S., Hussain S., Baeza-Squiban A. (2014). Carbon Black and Titanium Dioxide Nanoparticles Induce Distinct Molecular Mechanisms of Toxicity. Wiley Interdiscip. Rev. Nanomed. Nanobiotechnol..

[6] Zhao Y., Sun X., Zhang G., Trewyn B.G., Slowing I.I., Lin V.S.Y. (2011). Interaction of Mesoporous Silica Nanoparticles with Human Red Blood Cell Membranes: Size and Surface Effects. ACS Nano.

[7] Jiang L., Yu Y., Li Y., Yu Y., Duan J., Zou Y., Li Q., Sun Z. (2016). Oxidative Damage and Energy Metabolism Disorder Contribute to the Hemolytic Effect of Amorphous Silica Nanoparticles. Nanoscale Res. Lett..

[8] Rothen-Rutishauser B.M., Schürch S., Haenni B., Kapp N., Gehr P. (2006). Interaction of Fine Particles and Nanoparticles with Red Blood Cells Visualized with Advanced Microscopic Techniques. Environ. Sci. Technol..

[9] Purohit R., Vallabani N.V.S., Shukla R.K., Kumar A., Singh S. (2016). Effect of gold nanoparticle size and surface coating on human red blood cells. Bioinspired Biomim. Nanobiomater..

[10] Barkur S., Lukose J., Chidangil S. (2020). Probing Nanoparticle–Cell Interaction Using Micro-Raman Spectroscopy: Silver and Gold Nanoparticle-Induced Stress Effects on Optically Trapped Live Red Blood Cells. ACS Omega.

[11] Kwon T.W., Woo H.J., Kim Y.H., Lee H.J., Park K.H., Park S., Youn B.H. (2012). Optimizing Hemocompatibility of Surfactant-Coated Silver Nanoparticles in Human Erythrocytes. J. Nanosci. Nanotechnol..

[12] Al-Akhras M.A.H., Aljarrah K., Albiss B., Al-Khalili D. (2017). Influence of iron oxide nanoparticles (Fe_3_O_4_) on erythrocyte photohemolysis via photofrin and Rose Bengal sensitization. Photodiagnosis Photodyn Ther..

[13] Ziental D., Czarczynska-Goslinska B., Mlynarczyk D.T., Glowacka-Sobotta A., Stanisz B., Goslinski T., Sobotta L. (2020). Titanium Dioxide Nanoparticles: Prospects and Applications in Medicine. Nanomaterials.

[14] Li S.Q., Zhu R.R., Zhu H., Xue M., Sun X.Y., Yao S.D., Wang S.L. (2008). Nanotoxicity of TiO_2_ nanoparticles to erythrocyte in vitro. Food Chem. Toxicol..

[15] Bian Y., Chung H.Y., Bae O.N., Lim K.M., Chung J.H., Pi J. (2021). Titanium dioxide nanoparticles enhance thrombosis through triggering the phosphatidylserine exposure and procoagulant activation of red blood cells. Part. Fibre Toxicol..

[16] Liu K., Lin X., Zhao J. (2013). Toxic effects of the interaction of titanium dioxide nanoparticles with chemicals or physical factors. Int. J. Nanomed..

[17] Tsui S.M., Ahmed R., Amjad N., Ahmed I., Yang J., Manno F., Barman I., Shih W.C., Laua C. (2020). Single red blood cell analysis reveals elevated hemoglobin in poikilocytes. J. Biomed. Opt..

[18] Avsievich T., Popov A., Bykov A., Meglinski I. (2019). Mutual interaction of red blood cells influenced by nanoparticles. Sci. Rep..

[19] Ghosh M., Chakraborty A., Mukherjee A. (2013). Cytotoxic, genotoxic and the hemolytic effect of titanium dioxide (TiO_2_) nanoparticles on human erythrocyte and lymphocyte cells in vitro. Appl. Toxicol..

[20] Hadei M., Rabbani S., Nabizadeh R., Mahvi A.H., Mesdaghinia A., Naddafi K. (2021). Comparison of the Toxic Effects of Pristine and Photocatalytically Used TiO_2_ Nanoparticles in Mice. Biol. Trace. Elem. Res..

[21] Wood B.R., Kochan K., Marzec K.M., Ozaki Y., Baranska M., Lednev I., Wood B. (2020). Resonance Raman spectroscopy of hemoglobin in red blood cells. Vibrational Spectroscopy in Protein Research.

[22] Atkins C.G., Buckley K., Blades M.W., Turner R.F.B. (2017). Raman Spectroscopy of Blood and Blood Components. Appl. Spectrosc..

[23] Dybas J., Chiura T., Marzec K.M., Mak P.J. (2021). Probing Heme Active Sites of Hemoglobin in Functional Red Blood Cells Using Resonance Raman Spectroscopy. J. Phys. Chem. B.

[24] Wood B.R., Stoddart P.R., McNaughton D. (2011). Molecular Imaging of Red Blood Cells by Raman Spectroscopy. Aust. J. Chem..

[25] Jiang J., Oberdoerster G., Biswas P. (2009). Characterization of size, surface charge, and agglomeration state of nanoparticle dispersions for toxicological studies. J. Nanopart. Res..

[26] Zhang C., Lohwacharin J., Takizawa S. (2017). Properties of residual titanium dioxide nanoparticles after extended periods of mixing and settling in synthetic and natural waters. Sci. Rep..

[27] Shaikh S.F., Mane R.S., Min B.K., Hwang Y.J., Joob O. (2016). D-sorbitol-induced phase control of TiO_2_ nanoparticles and its application for dye-sensitized solar cells. Sci. Rep..

[28] Torres Filho I.P., Terner J., Pittman R.N., Proffitt E., Ward K.R. (2008). Measurement of hemoglobin oxygen saturation using Raman microspectroscopy and 532-nm excitation. J. Appl. Physiol..

[29] Wood B.R., Tait B., McNaughton D. (2001). Micro-Raman characterisation of the R to T state transition of haemoglobin within a single living erythrocyte. Biochim. Biophys. Acta Mol. Cell Res..

[30] Torres Filho I.P., Terner J., Pittman R.N., Somera L.G., Ward K.R. (2005). Hemoglobin oxygen saturation measurements using resonance Raman intravital microscopy. Am. J. Physiol. Heart. Circ. Physiol..

[31] Iavicoli I., Leso V., Fontana L., Bergamachi A. (2011). Toxicological effects of titanium dioxide nanoparticles: A review of in vitro mammalian studies. Eur. Rev. Med. Pharm. Sci..

[32] Shah S.N.A., Shah Z., Hussain M., Khan M. (2017). Hazardous effects of titanium dioxide nanoparticles in ecosystem. Bioinorg. Chem. Appl..

[33] De Matteis V., Cascione M., Brunetti V., Toma C.C., Rinaldi R. (2016). Toxicity assessment of anatase and rutile titanium dioxide nanoparticles: The role of degradation in different pH conditions and light exposure. Toxicol. Vitr..

[34] Yu Q., Wangm H., Peng Q., Li Y., Liu Z., Lia M. (2017). Different toxicity of anatase and rutile TiO_2_ nanoparticles on macrophages: Involvement of difference in affinity to proteins and phospholipids. J. Hazard. Mater..

[35] Kosmachevskaya O.V., Nasybullina E.I., Blindar V.N., Topunov A.F. (2019). Binding of Erythrocyte Hemoglobin to the Membrane to Realize Signal-Regulatory Function (Review). Appl. Biochem. Microbiol..

[36] Chu H., McKenna M.M., Krump N.A., Zheng S., Mendelsohn L., Thein S.L., Garrett L.J., Bodine D.M., Low P.S. (2016). Reversible binding of hemoglobin to band 3 constitutes the molecular switch that mediates O_2_ regulation of erythrocyte properties. Blood.

[37] De Rosa M.C., Carelli Alinovi C., Galtieri A., Scatena R., Giardina B. (2007). The plasma membrane of erythrocytes plays a fundamental role in the transport of oxygen, carbon dioxide and nitric oxide and in the maintenance of the reduced state of the heme iron. Gene.

[38] Ahlawat S., Kumar N., Uppal A., Gupta P.K. (2016). Visible Raman excitation laser induced power and exposure dependent effects in red blood cells. J. Biophotonics.

[39] Marzec K.M., Rygula A., Wood B.R., Chlopickia S., Baranska M. (2015). High-resolution Raman imaging reveals spatial location of heme oxidation sites in single red blood cells of dried smears. J. Raman Spectrosc..

[40] Menżyk A., Damin A., Martyna A., Alladio E., Vincenti M., Martra G., Zador G. (2020). Toward a novel framework for bloodstains dating by Raman spectroscopy: How to avoid sample photodamage and subsampling errors. Talanta.

[41] Dalai S., Pakrashi S., Suresh Kumar R.S., Chandrasekaran N., Mukherjee A. (2012). A comparative cytotoxicity study of TiO_2_ nanoparticles under light and dark conditions at low exposure concentrations. Toxicol. Res..

[42] You D.G., Deepagan V.G., Um W., Jeon S., Son S., Chang H., Yoon H.I., Cho Y.W., Swierczewska M., Lee S. (2016). ROS-generating TiO_2_ nanoparticles for non-invasive sonodynamic therapy of cancer. Sci. Rep..

[43] Ou G., Li Z., Li D., Cheng L., Liu Z., Wu H. (2016). Photothermal therapy by using titanium oxide nanoparticles. Nano Res..

